# Effect of structural repairs on the load rating and reliability of a prestressed concrete bridge

**DOI:** 10.1186/s43251-023-00087-0

**Published:** 2023-04-27

**Authors:** Marwan Debees, Furkan Luleci, F. Necati Catbas

**Affiliations:** Department of Civil, Environmental, and Construction Engineering, University of Central Florida, Orlando, FL 32816 USA

**Keywords:** System reliability, Component reliability, Prestressed bridge, Monte-Carlo Simulation, Structural repair

## Abstract

Prestressed girders often deteriorate over time due to environmental and man-made stressors, lowering the strength and serviceability of bridge structures. Although structural repairs are implemented to improve the load carrying capacity of the structure, the presence of numerous unknowns leads to high uncertainty in estimating the adequacy of repairs. For instance, the cross-section of the remaining strands, material properties, applied external loads, and workmanship assumptions made throughout the repair process introduce ambiguities when estimating the adequacy of the repairs. This study evaluates the efficiency of re-tensioning repairs of prestressed concrete bridge span girders. The repairs include field splicing, re-tensioning, of deteriorated or damaged strands by torquing a splicing coupler. The evaluation in this study considers component, system reliability, and load ratings while accounting for several uncertainties, such as structural repair, material properties, and external loads. This paper introduces an approach to account for prestressing strands damage and repair uncertainties while also accounting for other uncertainties. In this regard, five cases are studied: as-built, repaired, and three varying degrees of damage cases. First, the distribution for structural demand and capacity accounting for uncertainty in loads, material properties, and repair process is defined for each girder in the prestressed concrete bridge span. In doing so, Monte-Carlo simulation is employed to determine the distributions. Accordingly, the limit state function of the girders is defined from the obtained distributions. Then, the component reliability of each AASHTO (American Association of State Highway and Transportation Officials) Type II girder is calculated from the obtained reliability indices based on the determined limit state functions. Finally, a system reliability model of the span is developed from the component reliability of each girder. Some advantages and disadvantages of using component and system reliability index versus load rating in damaged and repaired prestressed concrete bridge girders are also discussed. Several critical conclusions are made regarding the uncertainties in structural repair, material properties and external loads, and their impact on the load rating and the component and system reliability of the prestressed concrete bridge structure girders.

## Introduction

In 1951, the Walnut Lane Memorial Bridge in Philadelphia was opened to traffic as the first long-span prestressed concrete bridge in the US (Zollman et al. 1992). Since then, prestressed concrete has become a very popular alternative for bridge engineers in the US and worldwide. Today, prestressed concrete girder bridges constitute approximately 50% of newly built bridges in the US (Aghagholizadeh and Catbas 2019). However, harsh environmental conditions pose a threat to structural integrity (Catbas et al. 2022). For instance, the chloride intrusion in the prestressed girders causes the strands and rebars to corrode, lowering the girder’s flexural strength. As the steel corrodes, its cross-section expands, causing the surrounding concrete to delaminate and eventually spall, reducing the serviceability of the girder and allowing more chloride penetration which accelerates the corrosion process. Therefore, timely and efficient structural repairs hold significant importance (Luleci et al. 2022). However, throughout the repairing process, the assumptions made by the engineers due to the presence of unknowns introduce numerous uncertainties. Multiple repair options for prestressed bridge are available, some of which are presented in the following. (1) Adding post-tensioning: The delaminated/spalled concrete is repaired in this method. External tensioning tendons are designed and installed on the beam to restore lost capacity. (2) Using internal re-tensioning splice: In this method, the delaminated/spalled concrete is removed, and the corroded steel sections are cleaned or removed. A splice is used to retention strands that are completely severed or with significant section loss. The concrete is then resurfaced to its original shape. (3) Using Fiber Reinforced Polymer FRP: The delaminated /spalled concrete is repaired in this method. The surface of the concrete is then prepared for wrapping. Fiber Reinforced Polymer is then applied on the prepared surface to restore or increase the capacity of the beams.

When these methods are field-implemented, the uncertainties of such application to repair the bridge is to be considered when the load carrying capacity, rating or reliability are to be determined. The authors have closely experienced such an application. The second repair method using re-tensioning splice, discussed in detail in this paper, has been a popular repair technique applied on bridges. There are several advantages and limitations of this repair. Some of the advantages are given as follows: it is the closest of the three methods to a return to print repair; it is a more straightforward repair design with vendor charts; it provides availability of material and trained contractors; and typically, girder cross section after the repairs has the dimensions as before the repairs (underside clearance not impacted). Furthermore, there are also limitations of the method, which are discussed in the next paragraphs.

The re-tensioning applied to the strands during the repairs is not transferred to the new concrete cover applied after the re-tensioning, potentially allowing the new concrete cover to crack. However, this could be mitigated by placing a live load (truck) on top of the girder during the repair process and removing this live load after the repair. The process transfers compression to the new concrete cover when removing the temporarily applied live load deflection. Furthermore, the new strand sections and splicing mechanism are susceptible to future corrosion due to the same environmental conditions which caused the original deterioration. In addition, corrosion can preferentially start at the interface of the parent and repaired concrete – also known as the halo effect (Krishnan et al. 2021). Resistance of the repaired sections to future corrosion could be improved significantly by utilizing cathodic protection. Cathodic protection may be achieved by using impeded galvanic anodes or metalizing by spraying zinc on the surface of the repaired section. Connectivity must be ensured for the sacrificial zinc to protect the strands and/or rebar steel. Moreover, this methodology can be sensitive to fatigue due to the repetitive nature of highway and railroad bridge loading, and they are not recommended when more than 15% of the strands are damaged (Harries et al. 2012; Zobel and Jirsa 1998; Gangi et al. 2017).

Lastly, vendor charts provide torque values needed to restore tension capacity based on strand grade and size. Strand splicing effectively restores the tensile strength of the strand to a value between 85 and 96% of its original value (85% is recommended as a conservative assumption) (Harries et al. 2012; Zobel and Jirsa 1998; Gangi et al. 2017). Nevertheless, the designer still makes certain assumptions, leading to uncertainties. These assumptions include the cross-section of the remaining strands, workmanship, and concrete mortar repair, which will fill the patch area with no voids. There are also uncertainties associated with calculating the flexural capacity of the original prestressing strand, such as material properties, workmanship, and tension losses. These are some of the real-life scenarios that cause uncertainties. The study herein investigates the uncertainties associated with the flexural strength limit state.

Reliability-based repair approaches are very important in accounting for such uncertainties (Estes and Frangopol 2001). Hence, this study investigates the impact of re-tensioning repairs of the prestressed concrete bridge girders on the component, system reliability, as well as load-rating values for the as-built, damaged, and repair scenarios while accounting for the uncertainties involved, such as structural repair, material properties and external loads.

### Background

It is pertinent to mention some of the reliability-based studies conducted in the literature. The authors in a study (Frangopol and Estes 1999) demonstrated that the reliability methods are beneficial and appropriate for optimizing the lifetime inspection and repair of structurally deficient structures to maintain an expected degree of lifetime performance for degrading bridges. In another study (Liang and Lan 2005), the authors investigated the corrosion of existing bridge concrete piles. The authors successfully found the structural joint failure probabilities of the bridge suffering carbonation, chloride ion ingress, and sulfate attack. Another study conducted a reliability analysis of an RC bridge to predict the likelihood and extent of cracking due to chloride attack considering material and deterioration parameters (Stewart and Mullard 2007). The authors found that the timing of the first repair for the bridge could be as soon as seven years for poor durability design specs and in excess of 120 years with great design specs. The authors concluded that the methodology could be utilized for a life-cycle cost analysis to optimize the maintenance approaches. The authors in Marsh and Frangopol (2008) built a reliability model by incorporating spatial and temporal variations of probabilistic corrosion rate from placed sensors on the RC bridge deck. The reliability model successfully estimated the service life of the RC bridge deck. In another study (Catbas et al. 2008), the authors investigated the reliability of the longest cantilever truss bridge in the US by incorporating sensorial data of wind pressure, dead load, traffic loads, temperature effects, and their combinations to minimize uncertainties. They demonstrated that the temperature variation influenced the developed reliability model the most. Another study investigated the reliability of a bridge considering creep, shrinkage, and corrosion using the CEB-FIP model (Guo et al. 2011). In that study, it is demonstrated that concrete creep and shrinkage play a significant role during the initial phases of structural deterioration, followed by a decrease in reliability due to a combination of creep, shrinkage, and corrosion. The authors in Liang et al. 2013 predicted the carbonation life of a concrete bridge using reliability and probability indices. The authors concluded that the methodology applied in that paper could be implemented to repair, strengthen, and demolish existing concrete bridge structures.

In another study (Kim et al. 2016), the authors introduced a method to evaluate the reliability of an in-service concrete highway bridge using resistance capacity degradation considering various traffic characteristics, corrosive environment, and crack formation. The study concluded that the reliability of that bridge is quite sensitive to traffic, corrosion, and crack factors which decreases the reliability level.

In this study, the authors investigate flexural strength. Traffic characteristics and a corrosive environment are of significant importance and will impact the overall reliability of the bridge. Since the research team observed the cross-section loss of strands during the repairs, the corrosive environment impact was accounted for by considering the appropriate cross-strand cross-section loss mean and Coefficient of Variation (COV), as discussed later in more detail. The available data is insufficient to consider the specific traffic characteristics of the case study bridge. Based on previous literature reviews, average values were utilized to account for the uncertainties in live load. Fine-tuning the mean and COV of live loads based on locally measured values could significantly impact the final reliability of the bridge. When measured traffic characteristics are available, they should be incorporated into the analysis. This study may be a basis for repair and reinforcement, life cycle cost analysis, and reliability evaluation of other bridges.

A recent study demonstrated that the health and performance of bridge components and overall systems could be assessed with reliability indices as weights (Inkoom and Sobanjo 2019). Using the importance weight index, the study found that the superstructure and substructure elements are critical to the survival of bridges, and any deterioration of the components of these subsystems significantly correlates with the overall bridge health and performance. The authors of that study recommended utilizing the health indices to assess the importance of bridge elements considering repair and maintenance. Another study discussed the critical concepts and methods for life-cycle reliability, risk, and resilience-based design and assessment of bridges and bridge networks considering independent and interacting hazards (Akiyama et al. 2020). One of the conclusions of that study is that more studies should further investigate the bridge performance under independent and interacting hazards considering uncertainty. More recently, a study (Parmiani and Orta 2022) assessed the safety of an RC bridge with varying reinforcement exposure lengths using the probability of failure, reliability indices, and deterioration coefficients. The authors of that study demonstrated that the exposure of reinforcement reduces the bending capacity, stiffness of the girders and, accordingly, the reliability indices. The change in stiffness is roughly proportional to the change in exposure length; however, the change in moment capacity varies differently. The load rating of bridges is also widely used in bridge engineering practice. The load rating of a bridge can be expressed as the ratio of the critical live load effect to the available capacity for a certain limit state. Load ratings represent a quantitative measure of identifying the need for load posting and/or bridge strengthening and making overweight-vehicle permit decisions. The final load rating represents the weakest point within the bridge in terms of the member and the failure mode. In this part of the paper, the bridge's load ratings are calculated by following (American Association of State Highway and Transportation Officials 2021, 2018) and using the models, which will be discussed in more detail. The load factors change according to the type of load rating, i.e., inventory or operating load rating. Equation (1) (American Association of State Highway and Transportation Officials 2021, 2018) gives the general formulation for the rating factor:

1 $$RF=\frac{C-{\gamma }_{DC}DC-{\gamma }_{DW}DW\pm {\gamma }_{p}P}{{\gamma }_{L}LL(1+IM)}$$

where RF is the bridge rating factor, C is the factored load carrying capacity, DC is the dead load of structural components, DW is the dead load of the wearing surface, P is permanent loads other than dead load., LL is the live load effect, IM is the impact factor (33% is used), and γ’s are the load factors defined for that bridge structure, which can be found in the AASHTO manual (American Association of State Highway and Transportation Officials 2021).

Akgul and Frangopol (Akgül and Frangopol 2004a) investigated a time-dependent relationship between the reliability-based analysis results, representing the future trend in bridge evaluation, and the load ratings for different types of bridges within an existing bridge network. Other studies also included reliability and load rating analysis of bridges considering different damage scenarios, particularly for steel and movable bridges. In these studies, multiple models are employed to incorporate uncertainty in the performance prediction of the bridges (Gokce et al. 2013, 2011; Catbas et al. 2013). There are other studies for field test data-based load rating analysis. One of the past studies investigated practical load rating methods for reinforced concrete T-beam bridges (Catbas et al. 2012).

Nowak and Rakoczy (Nowak and Rakoczy 2013) investigated treating load and resistance in the building process as random variables. By utilizing statistical parameters, i.e. bias factors and coefficient of variations, they categorized uncertainties in the resistance in the building process into three subsets: material, fabrication, and professional. More information about these is presented in the following sections of this paper.

Gokce et al. (Gokce et al. 2013) presented a structural identification implementation by means of a family of calibrated models for load rating and reliability prediction with the consideration of uncertainties. They utilized a parent and offspring models in their study of engineering concepts such as load rating of bridges and system-level reliability while considering uncertainties and the correlation of different components. They reported the system reliability of the movable bridge by using a series-system model. The system's reliability is predicted within bounds by perfectly correlated and independent safety margins. Results for the present time (t = 0) were a 4.45 reliability index when no correlation is assumed and 4.58 when a perfect correlation is assumed. A broader study was conducted more recently by Frangopol et al. (Frangopol et al. 2019; Frangopol and D M, Zhu B, Sabatino S. 2018) to investigate the AASHTO redundancy factor for steel and concrete bridge systems. The system reliability index is affected not only by the reliability of its components but also by other parameters, such as correlation among resistances of components and system arrangement, among others. Therefore, it is necessary to study the reliability of systems whose components are designed to have a consistent reliability level (Frangopol and D M, Zhu B, Sabatino S. 2018). The study investigates systems with multiple components having the same component reliability index while configured in different system reliability models (Series (S), Parallel (P), and Series–Parallel (S-P) (more information on system reliability modelling will be presented in the following sections of this paper). Components resistance correlations are considered by calculating the system reliability at the perfect correlation (correlation = 1), No correlation (correlation = 0) and 50% correlation (correlation = 0.5). For perfect correlation among failure modes of components existing, both the resistance and load effects have to be perfectly correlated. In many cases, it may be difficult to determine the real correlation of variables. As a result, one approach is to consider different correlation cases to determine the final outcome. As a results, Frangopol et al. created tables to predict the system reliability under different assumptions.

Here, this paper investigates a deteriorated bridge's load rating and component and system reliability before and after the repairs. The component reliabilities here are not all the same as in the previously mentioned study. Furthermore, as verified in the field, each girder component has deteriorated at a significantly different rate than the other components. This will become apparent as the actual damage and repair cases are presented in the subsequent sections of this paper.

Harries et al. (Harries et al. 2012) investigated collision damage and repair of prestressed concrete beams. In their research, published by the National Cooperative Highway Research Program (NCHRP), they summarize a survey for the State of Practice. Some o the findings are as follows: (1) Load capacity is the dominant consideration when selecting a repair method. Other considerations are the durability of the repairs, interruption of service, and time to repair. (2) There was no dominant repair method (the repair method is site-specific). However, strand splicing was specifically cited by half the respondents. (2) Most minor impact-related damage (concrete cracks and nicks; shallow spalls and scrapes not affecting tendons). (3) Many respondents did not specify how the “repair or replace” decision for impact-damaged prestressed girders is made. One jurisdiction indicated they repair girders with up to 2 ruptured strands and replace them if three or more strands are ruptured (no analysis is performed). Others conduct load rating analyses and perform repairs only as needed. (4) When load rating is conducted, the model is based on input from a visual inspection.

### Purpose and scope

Over the last few decades, reliability-based analysis and design methods have seen great attention in research and development as well as in industry by practicing engineers. It is widely accepted that as the reliability concept is further and better comprehended, less timely and accurate maintenance is achievable, enabling more efficient life-cycle management of bridge structures. As reliability concepts are further understood and developed, more efficient and accurate maintenance is achievable, enabling more effectual life-cycle management of bridge structures. After an extensive literature review, including but not limited to the studies mentioned above, the authors of this paper observed that there is room to investigate the impact of re-tensioning repairs of the prestressed concrete bridge girders on the component, system reliability, as well as load-rating values for the as-built, damaged, and repair scenarios while accounting for the uncertainties involved, such as structural repair, material properties and external loads. Particularly, it is critical to understand the adequacy of structural repairs, which introduces various uncertainties to the structure, as mentioned earlier. In this regard, five cases are studied: as-built, repaired, and three varying degrees of damage cases.

**Fig. 1:**
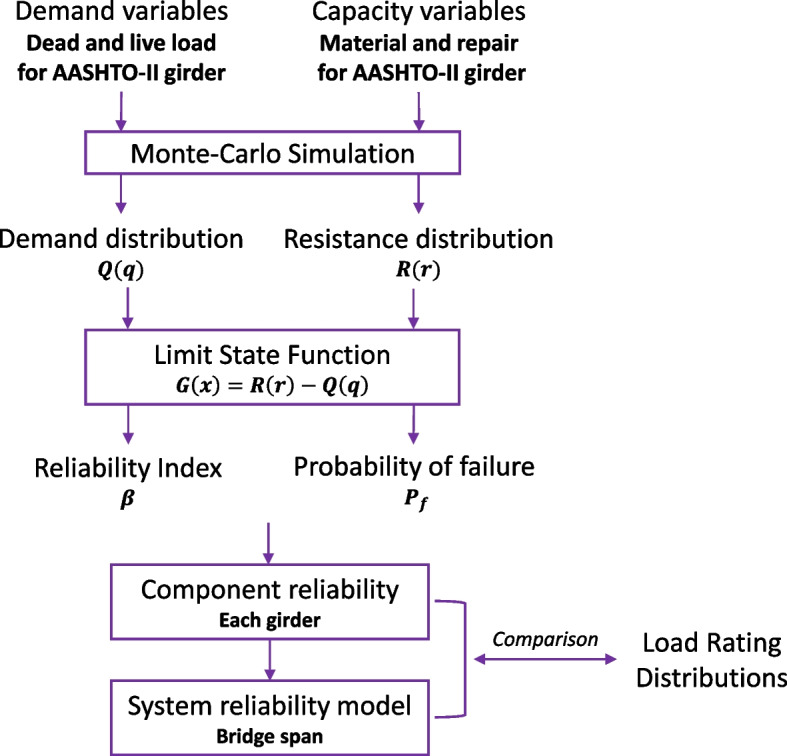
The scope of the study

The scope of this study is illustrated in Fig. 1. First, for each girder, the distribution characteristics of demand (dead and live loads) and capacity (material properties and repair process) for the AASHTO Type II girder are defined based on applicable codes and field inspection. Then, the distribution of structural demand, $Q(q)$, and structural capacity, $R(r)$, are obtained for each girder in a span of five girders using Monte-Carlo simulation. Subsequently, the limit state function, $G(x)$, is calculated in Eq. (2), which defines it as the boundary between the safety and failure region. Followingly, the reliability index, $\beta$, is computed in Eq. (3) to find the component reliability index of each girder in the span. Additionally, the probability of failure, ${P}_{f}$, is determined in Eq. (4). Then, the system reliability model of the span is developed using the obtained component reliability indices. Finally, some pros and cons of using component and system reliability index versus load ratings of damaged and repaired prestressed concrete bridge girders are discussed.

2 $$G(x)=R(r)-Q(q)$$

3 $$\beta = {\Phi }^{-1}{(1-P}_{f})$$

4 $${P}_{f}=Q\left(q\right)> R(r)$$

## The rationale for using system reliability

The typical load rating (LR) calculation procedure accounts for the portion of the dead and live loads applied on each bridge girder by dividing the dead load and using a distribution factor for the live load. Then, the AASHTO Load and Resistance Factor Design (LRFD) Bridge Design Specifications code uses a component reliability-based approach to load-rate the superstructure using the predicted load and resistance of the most critical girder. Yet, in reality, bridge girders under the same span do not behave independently. For example, in the case of the studied bridge structure in this paper, the cast-in-place composite bridge deck and the secondary diaphragm introduce a mechanism for load redistribution amongst the five AASHTO Type II girders. Therefore, in most cases, the redundancy and ductility factors cause the component-based design to be on the conservative side. When the load in a critical component approaches the ultimate value, other components can take additional loads and prevent failure (Nowak 2004). A system reliability approach is considered to quantify this partial collaboration mechanism of bridge components. A system model represents a bridge as an idealized assembly of components with the potential for whole structural failure (Frangopol et al. 2019). Depending on how the components are arranged in the system reliability model, a failure occurs when some or all components reach their failure limit state. For instance, system reliability with all components arranged in a series model fails when any of its components reach their limit state failure. A statically determined truss bridge might have components and connections that are not redundant. This non-redundancy in structural components can lead to a systematic failure when one component or connection fails. On the other hand, when the system is modelled in parallel, a failure occurs when all the parallel components reach their limit state failure. A cable-stayed bridge can be modelled as a parallel system where the system fails when all primary carrying components of spans fail.

**Fig. 2:**
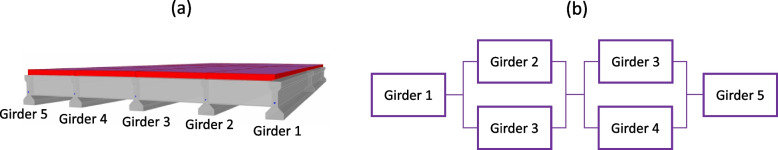
(**a**) Typical bridge span, (**b**) Assigned system reliability model for the span

Identifying an appropriate system model type to represent a specific bridge may require considerable engineering judgement (Frangopol et al. 2019). Given the type, design, and age of the studied bridge in this paper, which is explained in the next section, a Series Parallel (S-P) model is assigned to represent the system reliability of the span of the bridge. This particular model uses an S–P system, where Girder 2 – Girder 3, and Girder 3 – Girder 4 are parallel connected (2 Parallel), and Girder 1 and Girder 5 are connected as series to the rest of the system model. As a result, the bridge superstructure fails when one external girder or two adjacent internal girders fail. Figure 2 (a) and Fig. 2 (b) show the typical span of the bridge and the designed system model, respectively.

## Case study bridge

**Fig. 3:**
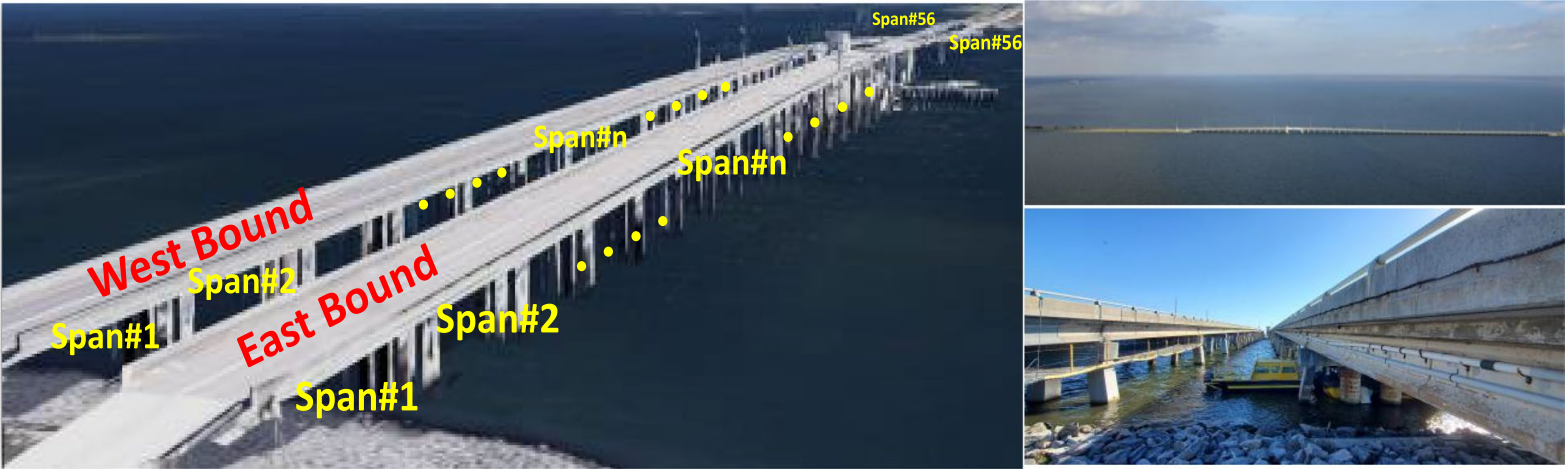
Multi-span prestressed concrete highway bridge

The bridge structure studied in this paper is a multi-span bascule bridge built in 1964 as two separate bridges, Eastbound and Westbound. Each bridge consists of 53 prestressed I-Beam spans with 52 ft each, two flanking spans, and a steel double leaf bascule main span (total of 56 spans), 129.5 ft between trunnion centers. The simple span bridge girders are AASHTO Type II Girders spaced at 6 ft 6 in on center with a 7 in cast-in-place deck and wearing surface. The bascule span is a steel span with special structural configurations. The multi-span prestressed concrete highway bridge is shown in Fig. 3. The span studied in this study is Span#1 on East Bound, though the spans of the bridge are structurally near identical based on their geometric and material properties as well as positions (Luleci et al. 2022; Dong et al. 2020). Thus, any other span could also be used in this study.

**Fig. 4:**
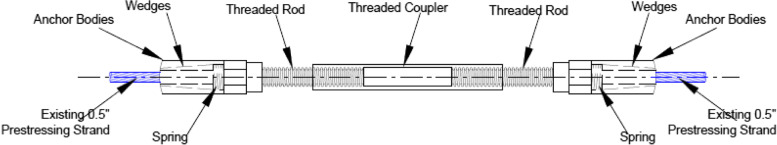
Prestressed strand splice repair

Some of the bridge girders and piles have undergone structural repairs in the past few years due to strands corrosion which led to concrete delamination and spalling. The method for girder repair included removing delaminated and spalled concrete and cleaning or removing corroded steel sections. A splice is used to re-tension strands completely severed or with significant section loss. A cathodic protection system is then employed, and the concrete cover of the girder is resurfaced to its original shape. Prestressed strand splice repair is illustrated in Fig. 4.

## Methodology

### Identification of the demand distribution

**Fig. 5:**
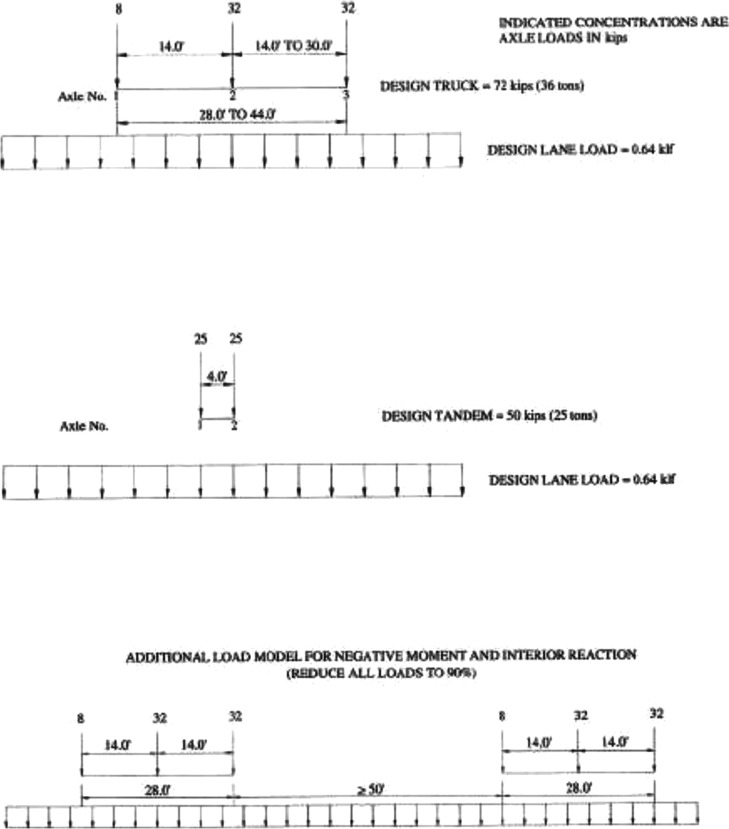
LRFD Design Live Load (HL-93) (American Association of State Highway and Transportation Officials 2018)

Bridge dead load and AASHTO HL-93 loading are applied on the bridge span to generate demand (load) distributions. HL-93 is a live design load adopted by American Association of State Highway and Transportation Officials (2021); American Association of State Highway and Transportation Officials 2018) to envelop the response of other truck loads. Figure 5 illustrates the loading cases included in HL-93: design truck plus design lane load or design tandem plus design lane load. For the average daily truck traffic of 5000, the bias factor of measured truck load responses to convert to 75-year maximum moments is from 1.3 to 1.5, and the coefficient of variation is 0.12 (Nowak and Rakoczy 2013). In this study, we apply a bias of 1.4 and a coefficient of variation of 12%. While the COV is utilized in reliability and load rating calculations, the bias factor is only utilized in the reliability calculations. Load rating calculations follow AASHTO equations which already include factors such as Live Load Factor (1.75 for inventory and 1.35 for operating), which makes the use of another bias factor not necessary. Bias factors are only used for reliability, not load rating calculations for all input parameters. An Impact Factor of 33% is applied (American Association of State Highway and Transportation Officials 2021).

Initial values for dead load and wearing surface were computed using information from the bridge files. All components of dead load can be treated as normal random variables (Nowak and Rakoczy 2013). The bias factor (ratio of mean-to-nominal) value of the dead load, *λ* is 1.05 and the (Coefficient of Variation) COV is 0.10 for cast-in-place concrete, and *λ* is 1.03 and the COV is 0.08 for precast concrete (Nowak and Rakoczy 2013). For the weight of asphalt, a mean value bias of 1.05 and a COV of 25% are applied (Nowak 2004).

### Identification of the variables for resistance distribution

Two distinguished sources of uncertainties were considered when calculating the capacity term distribution. The first source of uncertainties stems from the inherent dimension and material properties distribution. The second source of uncertainties stems from the deterioration and performed repairs.

#### Inherent dimension and material properties distribution

While there are uncertainties in all the dimensions and material properties included in calculating the capacity term, some of these uncertainties have a less significant contribution to the final capacity value than others. For instance, an increase or a decrease by a fraction of an inch to the girder span of 52 ft would not have the same contribution as a change in the depth of prestressing strands. Therefore, the uncertainty in the change of depth of prestressing is addressed separately. Lu et al. reported the effective rebar depth in concrete to have a mean value bias of 1.0 and a coefficient of variation of 2% (Nowak 2004; Lu et al. 1994).

Nowak and Rakoczy (Nowak and Rakoczy 2013) used Eqs. (5,6 and 7) to calculate the final Resistance R, the mean of the final resistance and the coefficient of variation of the final resistance, respectively. The load carrying capacity or resistance, R, is considered a product of the nominal resistance, Rn and three parameters: strength of the material, M, fabrication (dimensions) factor, F, and analysis (professional) factor, P.

This paper addresses the strength of material uncertainties, M, and deterioration and repair process uncertainties using Monte Carlo Simulation. Dimensions and professionalism factors are utilized to address overall dimensions and professional uncertainties.

5 $$R={R}_{n}MFP$$

The statistical parameters of the fabrication and professional factors vary for λ (bias factor) between 1.0–1.05 and COV between 0.01–0.04 for the dimensions; and for λ between 1.0–1.05 and COV between 0.04–0.06 for the professional factors (Nowak and Rakoczy 2013). Average values of λ and COV are used in this paper for both fabrication and professional factors.

6 $${\mu }_{R}={{R}_{n}\mu }_{M}{\mu }_{F}{\mu }_{p}$$

7 $${COV}_{R}=\sqrt{{{COV}_{M}}^{2} {+ {COV}_{F}}^{2} {{+ COV}_{P}}^{2}}$$

The distribution of concrete strength can be treated as the normal distribution when its scope is not too wide (Dayaratnam and Ranganathan 1976). Sakai et al. showed that the distribution pattern of the tensile strength of structural steel could be approximated by both normal and log-normal distribution (Sakai et al. 1997). The bias and COV of concrete compressive strength are taken as 1.14 and 14%, while the bias and COV of the prestressing steel strength are taken as 1.04 and 2% (Nowak and Rakoczy 2013).

#### Uncertainties due to deterioration and performed repairs

The girder repair method included the removal of delaminated/spalled concrete and the cleaning or removing of corroded steel sections. A splice is used to retention strands that are completely severed or with significant section loss. A cathodic protection system is then employed, and the concrete is resurfaced to its original shape.

The remaining cross-section of the strands is a key factor in calculating the capacity of the girder. There is a great deal of uncertainty in assuming this remaining cross-section. The repair design documents for the case study bridge define “significant” cross-section loss in a strand as equal and more than 25% section loss. Any strand found to have more than 25% section loss would be severed, and a new strand section would be added and retensioned. Thus, assuming all remaining strands have less than 25% section loss after the repairs is a safe assumption. It is also reasonable to assume that strands exposed during the clearing of spalled/delaminated concrete endure some section loss due to corrosion. Prestressing strands typically start corroding when the chloride concentration in the surrounding concrete reaches the corrosion threshold limit at a depth of the strands. This paper assumes that all exposed strands and the two adjacent strands endure section loss due to corrosion. The following assumptions are made for all the studied cases, as introduced in the next section (Cases studied section):

- A strand in the as-built condition has 100% of its cross-section (no section loss).
- A strand is identified as significantly damaged if it has a section loss between 25 and 100% for the 3-sigma population.
- All exposed strands plus two adjacent strands, except those identified as significantly damaged, have section loss between 0 and 25% for the 3-sigma population.
- Repaired strands continue to have section loss between 15 and 25% for the 3-sigma population. (15% is based on previous literature recommendations (Harries et al. 2012; Zobel and Jirsa 1998; Gangi et al. 2017).

### Cases studied

#### As-built case

**Fig. 6:**
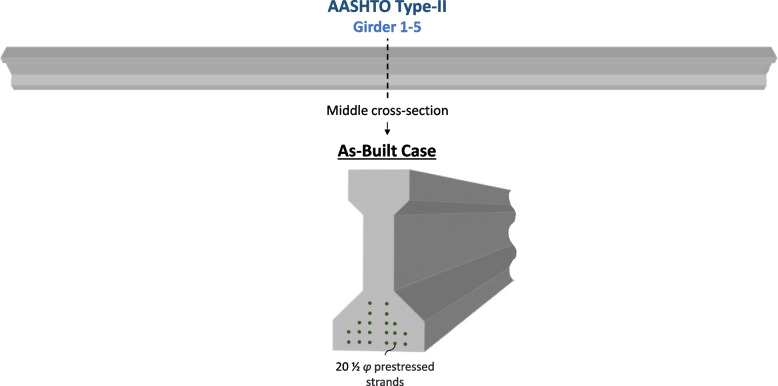
As-built case of the girder in the bridge span

The superstructure conditions are assumed to be as-built, with no section loss. Equation (8) denotes the mean value of the cross-section of the strands. As-Built Case is visualized in Fig. 6.

8 $${\mu }_{As-Built}={A}_{s\left(total- As-Built\right)}$$

#### Repaired case

**Fig. 7:**
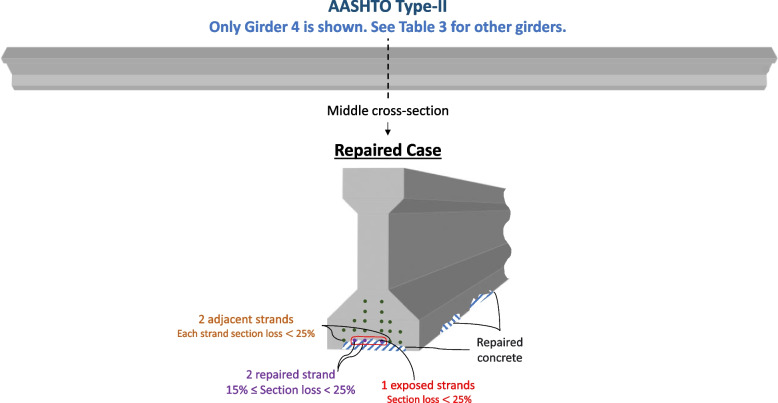
Repaired Case of Girder 4 in the bridge span (see Table 3 for other girders): 3 exposed strands, including one that was repaired (spliced). The spalled concrete is also resurfaced

An actual span repair case is considered. The number of repaired strands for each girder is shown in Table 1. For example, Girder 2 had three exposed strands, including one that had more than 25% section loss and, as a result, was repaired, and two with section loss of less than 25% were not repaired. Two additional adjacent strands are also assumed to have less than 25% section loss. Repaired Case is visualized for Girder 2 in Fig. 7.

**Table 1:** The number of strands exposed and repaired in each girder

Girder	# Strands Repaired	# Strands Exposed
1	3	4
2	1	3
3	0	1
4	2	3
5	6	6

Since this case considers the span after the repairs are already completed, no strand is expected to have more than 25% section loss. From field observation, some strands had section loss that is less than 25%. According to the repair design documents, these strands were properly cleaned and protected from future corrosion by utilizing cathodic protection. However, no splicing was performed since the section loss was less than 25%. This engineering approach is considered to avoid severing good strands with minor section loss. Severing prestressing strands in the field is a critical repair process that is done only when necessary and requires the approval of the responsible engineer. While insignificant, these minor strand section losses still impact the flexural capacity of the superstructure member. There is uncertainty in estimating how much this impact is since the exact amount of minor section loss is not known. It is reasonable to assume that the strands within and around the repaired area are more likely to have these minor section losses than strands in other intact areas. Therefore, a new approach is suggested in this paper to account for such uncertainty. All exposed strands plus the two adjacent strands are assumed to endure section loss due to corrosion. Based on the repair design documents, field observations and engineering judgment, the maximum cross-section loss in the 3-sigma population is assumed to be 25%. This is in line with the repair design documents, which require replacing strand sections with section loss greater than or equal to 25%. The minimum section loss in the uncertainty analysis depends on whether or not the strand received splicing repairs. Previous studies (Harries et al. 2012; Zobel and Jirsa 1998; Gangi et al. 2017) recommended the effective number of strands restored by strand splicing to be taken as 0.85 of the number of the original strands repaired. Following these recommendations, repaired strands are assumed to have at least 15% section loss. In summary, for repaired girders, spliced strands are assumed to have section loss ranging between 15 and 25%, while exposed strands did not receive splicing repairs (plus an additional two adjacent strands) are assumed to have section loss between 0 and 25%. Table 1 shows the number of strands that were exposed and repaired during the repairs of each of the five girders. Equation (9 and 10) utilize these values to calculate the mean and standard deviation values of each girder’s remaining cross-section of strands, where ${\mu }_{R}$ is the mean value of the remaining strands' cross-section area after the repairs, ${A}_{s\left(one strand- As-Built\right)}$ is the area of steel in one strand in the as-builts (0.153 in^2^), ${A}_{s\left(total- As-Built\right)}$ is the total area of prestressing strands per the as-builts ($0.153x20=3.06$ in^2^), $\sum \mathrm{exp}$ is the total number of strands exposed during the repair process, ∑rep is the total number of strands which received splice repair, and ${\sigma }_{R}$ is the standard deviation for the remaining strands' cross-section area after the repairs. The results are shown in Table 2.

**Table 2:** Mean and standard deviation values of remaining strands cross section after the repairs

Parameter Name	Symbol	Units	Nominal Value	Mean	Bias	SD	COV	Reference
G1 (Total cross-section area of strands for Girder 1)	Aps	in^2^	3.06	2.911	0.951	0.021	0.71%	Estimated based on number of strands exposed and repaired
G2				2.953	0.965	0.026	0.87%	
G3				3.00	0.981	0.019	0.64%	
G4				2.941	0.961	0.020	0.67%	
G5				2.838	0.927	0.020	0.7%	

9 $${\mu }_{R}={A}_{s\left(total- As-Built\right)}-{A}_{s\left(one strand- As-Built\right)}[\left(\sum \mathit{exp}-\sum \mathit{rep}+ 2)* \frac{0.25}{2}\right)+\left(\sum rep*\frac{0.15+0.25}{2}\right)]$$

10 $${\sigma }_{D}=\sqrt{\begin{array}{c}{\left(\sum exp-\sum rep+2)*\frac{0.25-0.125}{3}*{A}_{s\left(one strand- As-Built\right)}\right)}^{2}+\\ {(\left(\Sigma rep\right)*\frac{0.25-0.2}{3}*{A}_{s\left(one strand- As-Built\right)})}^{2}\end{array}}$$

#### Damage Case-I

**Fig. 8:**
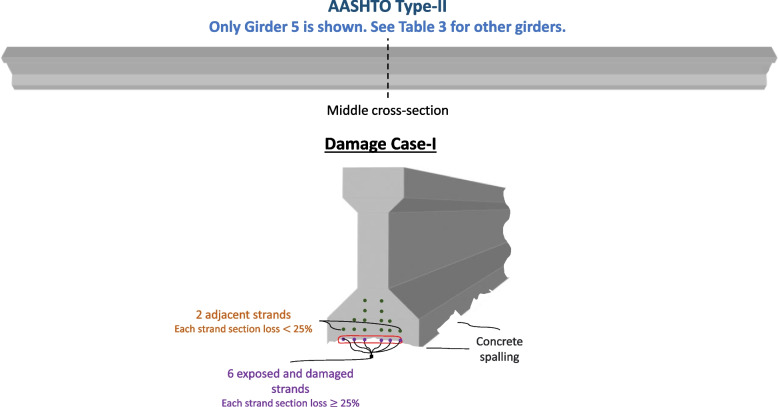
Damage Case-I of Girder 5 in the bridge span (see Table 3 for other girders): 6 damaged strands are present, each having section loss equal to or more than 25%. Concrete spalling is also present in the girder. Two adjacent strands are assumed to have less than 25% section loss

Three damage cases are investigated. The first damage case (Damage Case-I) considers the actual damage of the repaired span before the repairs are completed. A damaged strand is a strand that was exposed during the repairs and found to have a section loss equal to or more than 25%. All remaining exposed strands plus two adjacent strands are assumed to have section loss between 0 and 25%. Table 3 shows the number of damaged and exposed strands in each girder. For example, Girder 5 has six exposed and damaged strands with equal or more than 25% section loss. Two adjacent strands are assumed to have less than 25% section loss. Damage Case-I is visualized in Fig. 8.

**Table 3:** The number of damaged and exposed strands in Damage Case-I

Girder	# Strands Damaged	# Strands Exposed
1	3	4
2	1	3
3	0	1
4	2	3
5	6	6

Estimating the mean and standard deviation of a damage case is more complicated than the previous cases. In the repaired case, no strand has a section loss greater than 25% (repair design documents). On the other hand, in a damage case, there is insufficient data to predict the range of uncertainty before the repairs. However, some factors could help in the process. For example, it is known that the repaired strands had significant section loss before the repairs, which was equal to or greater than 25%. From field observation, this loss ranged from 25 to 100%. It is also known from the field observation that many of the exposed strands, which did not have enough section loss to warrant a repair, still had some section loss. This loss ranged from 0 to less than 25%. Conservatively, two additional strands adjacent to the exposed strands are also assumed to experience some section loss. The 3-sigma population for the exposed and damaged strands is assumed to have between 25 and 100% section loss. The 3-sigma population for the remaining exposed strands plus two is assumed to have between 0 and 25% section loss. Based on the repair design requirements, field observation, and engineering judgment, Eqs. (11 and 12) are formulated assuming a normal distribution, where ${\mu }_{D}$ and ${\sigma }_{D}$ are the mean and standard deviation of the cross-section area of the remaining strands, and $\sum \mathrm{D}$ is the total number of damaged strands which later received re-tensioning repairs.

11 $${\mu }_{D}={A}_{s\left(total- As-Built\right)}- {A}_{s\left(one strand- As-Built\right)}*\Sigma D*\frac{1+0.25}{2}-{A}_{s\left(one strand- As-Built\right)}*\left(\Sigma exp+2-\Sigma D\right)*\frac{0.25}{2}$$

12 $${\sigma }_{D}=\sqrt{\begin{array}{c}{\left(\Sigma D*\frac{1-0.625}{3}*{A}_{s\left(one strand- As-Built\right)}\right)}^{2}+\\ {(\left(\Sigma exp+2-\Sigma D\right)*\frac{0.25-0.125}{3}*{A}_{s\left(one strand- As-Built\right)})}^{2}\end{array}}$$

#### Damage Case-II

**Fig. 9:**
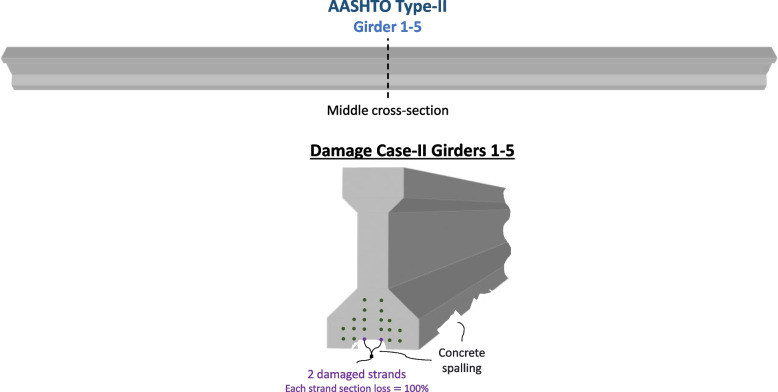
Damage Case-II of Girder 1–5 in the bridge span: 2 damaged strands are present, each strand having section loss equal to 100%. Concrete spalling is also present in the girder

The second damage case (Damage Case-II) considers a hypothetical case where every girder experiences 100% section loss for 2 of its 20 strands in the lower strand layer. This case is being investigated since Girder 3 did not experience significant damage that warranted re-tensioning repairs in the actual repair span. The section loss, in this case, is assumed to be deterministic, and the uncertainty in the remaining cross-section area is addressed using the overall reliability dimension factor. The damage in this hypothetical case is unique to the other damage cases. Damage Case-II is visualized in Fig. 9.

#### Damage Case-III

**Fig. 10:**
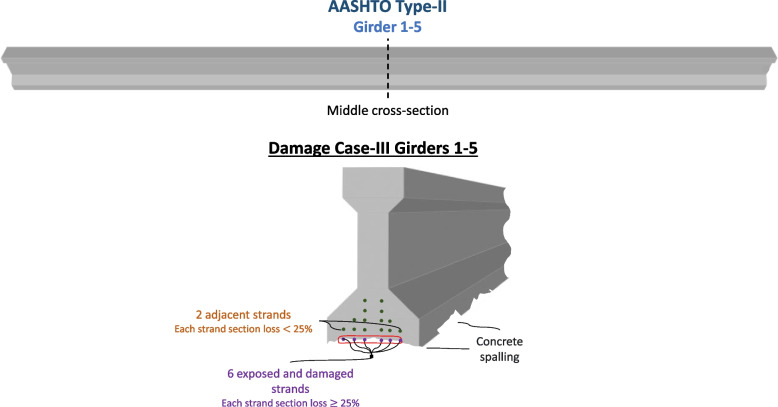
Damage Case-III of Girder 1–5 in the bridge span: 6 exposed and damaged strands present, each strand having section loss equal to or more than 25%. Concrete spalling is also present in the girder. Two adjacent strands in the second layer are assumed to experience section loss of less than 25%

The third damage case (Damage Case-III) considers a hypothetical case where every girder experiences similar damage to the damage experienced by Girder 5 in Damage Case-I. For every girder, the six lower layer strands have section loss equal to or more than 25% and two strands of the second layer on the sides experience section loss lower than 25%. This case is expected to result in a significantly lower load-rating value and reliability index because Girders 2, 3, and 4 have higher distribution factors than Girder 5. Since one of the girders, Girder 5, has experienced this section loss in real life, the authors believe it is appropriate to investigate what similar damage would do to other girders. Damage Case-III is visualized in Fig. 10.

### Load rating calculations

Both inventory and operating load ratings are investigated for the flexural limit state at mid-span. The inventory rating is the lower of the two ratings. It represents the load level at which a structure is safe for an infinite period of time. In contrast, the operating rating is the absolute maximum load that should be allowed on the bridge under any circumstances (Estes and Frangopol 2005). The load rating (LR) equation was given in Eq. 1 and can be found in Inkoom and Sobanjo (2019).

**Fig. 11:**
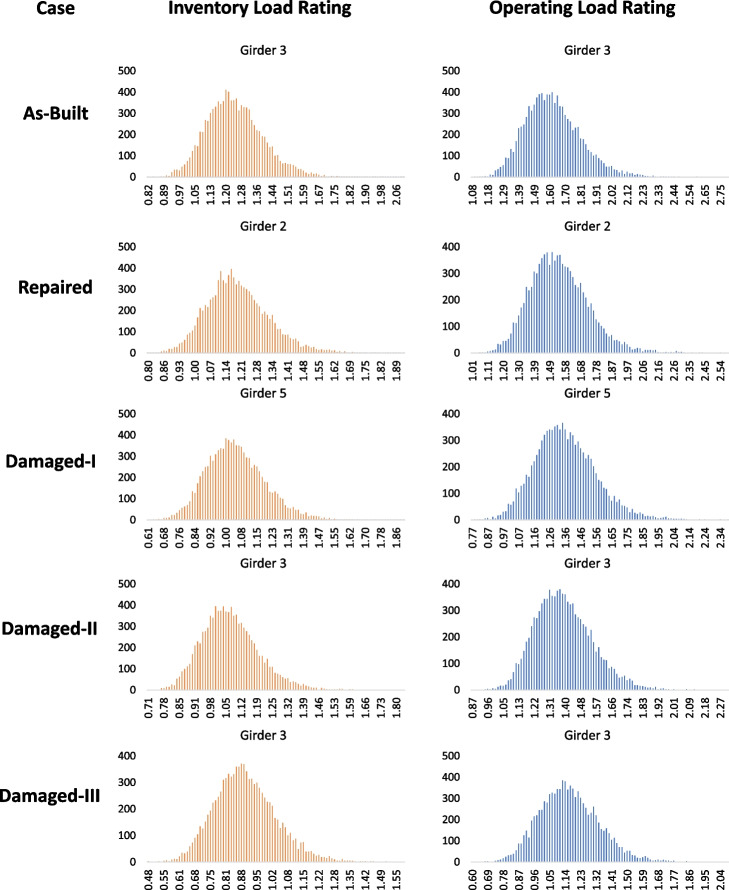
The inventory and operating load rating distributions for different cases for the controlling girders as shown in Table 4

The dead loads are identified from the bridge documents based on the as-builts to calculate the load rating. Maximum live loads are computed using AASHTO HL-93 truck loading conditions. Load Factors and Impact Factors are defined in the AASHTO Code (American Association of State Highway and Transportation Officials 2021). While operating and inventory load ratings distributions are calculated, the operating load rating is compared and contrasted with reliability calculations. This is consistent with some of the previous publications by others (Gao 2019; Akgül and Frangopol 2004b). Operating load rating could easily be substituted by inventory rating by simply switching the appropriate AASHTO factors if desired. Accordingly, the mean values for the HL-93 operating load rating are calculated and given in Table 4. The lowest mean operating load rating values are bolded for each girder for the respective cases and taken as the controlling girder of the whole span system. Additionally, both the inventory and operating load rating distributions for the different cases are given in Fig. 11.

**Table 4:** Mean values for HL-93 operating load rating (^a^controlling girder)

Case	Girder 1 (G1)	G2	G3	G4	G5	Controlling Load Rating as % of As-Built Load Rating
As-Built	1.89	^a^**1.61**	^a^**1.61**	^a^**1.61**	1.89	100%
Repaired	1.79	1.53	1.56	^a^**1.52**	1.70	94%
Damage-I	1.59	1.48	1.56	1.42	^a^**1.35**	84%
Damage-II	1.62	^a^**1.38**	^a^**1.38**	^a^**1.38**	1.62	86%
Damage-III	1.34	^a^**1.15**	^a^**1.15**	^a^**1.15**	1.32	71%

### Reliability calculations

**Fig. 12:**
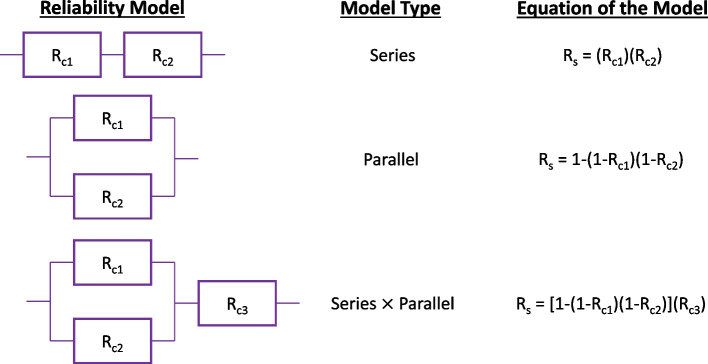
The reliability model, model type, and equations of the models to develop the system reliability model (the component is denoted as R_c_)

System reliability models are typically generated by developing a relationship between the reliability of its components. These component reliabilities are connected in a series, parallel, or mixed configuration. The reliability of a component is simply referred to as R_c_ in this study, where R_c_ is 1 – F_c_ and F_c_ is the component probability of failure. Likewise, R_s_ is referred to as the system reliability, where R_s_ is 1 – F_s_, and F_s_ is the system probability of failure. Figure 12 illustrates system reliability equations based on the component reliability configurations. The system reliability model of the superstructure developed in this study is shown in Fig. 2 (b). The model is developed based on the studied bridge's type, design, and age, alongside the best engineering judgment. Furthermore, this paper does not consider the correlation between component reliability variables. In order to determine the impact of different correlations between components on the system reliability, previous publications (Akgül and Frangopol 2004a; Nowak and Rakoczy 2013; Frangopol et al. 2019) have calculated the system reliability at both perfect correlation and no correlation by utilizing RELSYS (Reliability of Systems) (Estes Allen 1998). In these studies, the reliability of any structure that can be modelled as a combination of series and parallel systems can be calculated. As there is not enough data available to calculate the correlation between the components' failure modes considered in this paper, it is safe to assume that a perfect correlation does not exist. Furthermore, based on the significantly varying degrees of deterioration between different girders, the researchers believe the resistance correlation may be closer to no correlation than a perfect correlation. As the scope of this paper is not to study the impact of correlation on the system reliability but rather a practical evaluation of splicing repairs on the capacity of a deteriorated bridge, no correlation assumption was utilized for all cases. Similarly, before and after repairs are evaluated using the same assumption of no correlation. Future publications may further investigate computing system reliability by using different correlation assumptions.

The component reliability of Girder 1 in this study is denoted as R_c1_. Similarly, the rest of the girders are denoted as R_c2_, R_c3_, R_c4_, and R_c5_, and the system reliability of the whole superstructure span is represented as R_s_. To calculate R_s_, Eq. (6) is used.

13 $${R}_{s}={R}_{c1}(1-(1-{R}_{c2})(1-{R}_{c3})(1-\left(1-{R}_{c3}\right)\left(1-{R}_{c4}\right)){R}_{c4}$$

To compute the system reliability, the component reliability index for each girder is first calculated by employing Monte-Carlo Simulation to account for the material, load, and process uncertainties. The reliability index for each component is calculated as shown in Eq. (14), where $\mathrm{\beta c}$ is the component reliability index, $\mathrm{Ec}\left(\mathrm{R}\right)$ is the mean value of the girder resistance, $\mathrm{E}(\mathrm{P})$ is the mean value of the load on the girder, $\mathrm{COV}\left(\mathrm{R}\right)$ is the coefficient of variation of the girder resistance, and $\mathrm{COV}\left(\mathrm{P}\right)$ is the coefficient of variation of the load on the girder. Fc and Rc are computed from βc using computer software. Equation (13) calculates the final system reliability for each case. Accordingly, in Table 5, the component reliability of each girder for each case and the corresponding system reliability values are given.

**Table 5:** The component reliability values of each girder and the system reliability values for each case are shown (^a^controls the component reliability)

Cases	Girder 1 (G1)	G2	G3	G4	G5	System
As-Built	6.31	^a^**5.29**	^a^**5.29**	^a^**5.29**	6.29	6.19
Repaired	5.79	4.89	5.08	^a^**4.85**	5.59	5.54
Damage-I	5.01	4.70	5.09	4.39	^a^**3.61**	3.61
Damage-II	5.57	^a^**4.53**	^a^**4.53**	^a^**4.53**	5.58	5.45
Damage-III	3.45	^a^**2.52**	^a^**2.52**	^a^**2.52**	3.46	3.23

14 $$\beta c=\frac{Ec\left(R\right)-E(P)}{\sqrt{{(Ec\left(R\right)COV\left(R)\right)}^{2}{+(Ec\left(R\right)COV\left(P)\right)}^{2}}}$$

Table 6 illustrates the probability of failure for each case when calculated based on the component reliability of critical members versus the probability of failure based on system reliability. It can be seen that when components are connected in parallel, the probability of failure values when considering the component reliability are generally higher than when considering the system reliability. Since the system reliability accounts for the collaboration between the girder components (it considers the redundancy in the system), the probability of failure values based on system reliability values are lower. However, the system reliability decreases if all components are connected in series. This is because, in a series configuration, the system fails if any of its components fail, while in a parallel configuration, all components have to fail for the system to fail. In the case of the S-P model, such as the one considered in this paper, the increase or decrease in system reliability, when compared to component reliability, depends on the location of the controlling member and the reliability values. This could be seen in the Damage-I case, where the system reliability is not higher than the component reliability of Girder 5. It is worth noting that Girder 5 is connected to the rest of the system in a series configuration.

**Table 6:** Probability of failure based on critical girder component reliability versus probability of failure based on system reliability

	Probability of Failure P_f_	
	Based on component reliability	Based on system reliability
As-Built	7.45 × 10^–8^	3.36 × 10^–10^
Repaired	5.50 × 10^–7^	1.60 × 10^–8^
Damaged-I	1.44 × 10^–4^	1.45 × 10^–4^
Damaged-II	3.22 × 10^–6^	2.66 × 10^–8^
Damaged-III	5.81 × 10^–3^	6.38 × 10^–4^

### Evaluation of the results

**Fig. 13:**
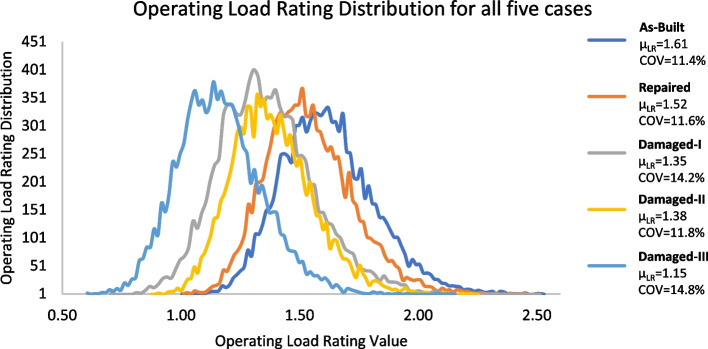
The HL-93 operating load-rating distribution for all five the cases is in one chart. The mean operating load rating (µ_LR_) and Coefficient Of Variation (COV) values are given for each case

**Fig. 14:**
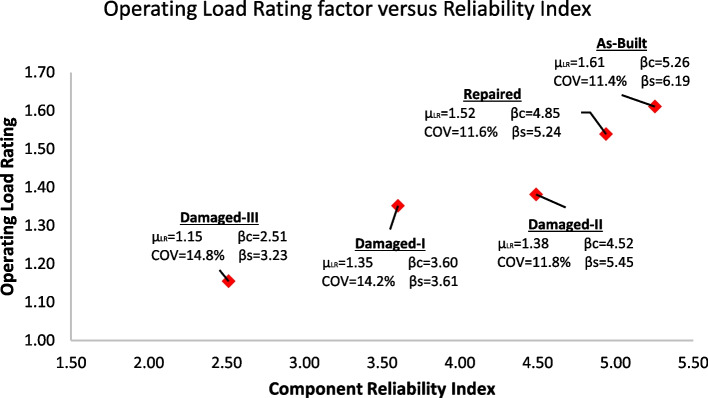
The mean of HL-93 operating load-rating versus component reliability index of the cases. The mean operating load-rating (µ_LR_), component reliability index (β_c_), system reliability index (β_s_), and Coefficient Of Variation (COV) values are given for each case

Figure 13 illustrates the combination of the operating load rating distributions for all five cases in one chart, while Fig. 14 provides the relationship between the operating load rating and the reliability index in each of the five cases. The first important observation is that performing repairs significantly improves reliability and load rating. However, they do not completely return to as-built conditions. This is due to the remaining cross-section loss and the higher uncertainties in a repaired section than in a new as-built section. Previous literature (Harries et al. 2012; Zobel and Jirsa 1998; Gangi et al. 2017) recommended using a deterministic value of a sufficient number of strands, restored by splicing, to be 85% of their original number. The recommended 85% was mainly associated with restored capacity and not specifically for additional cross-section loss due to corrosion deterioration. This study investigated the additional uncertainty in section loss in the spliced and exposed strands due to previous corrosion. It is also observed that the coefficient of variation is higher for the damaged cases. It should be indicated that this variation can be higher if more deterioration effects and uncertainties in future loadings are considered when bridges are rated not only for the given loads (e.g. HL 93) but also for data (e.g. weigh-in-motion/WIM) based operating traffic.

**Fig. 15:**
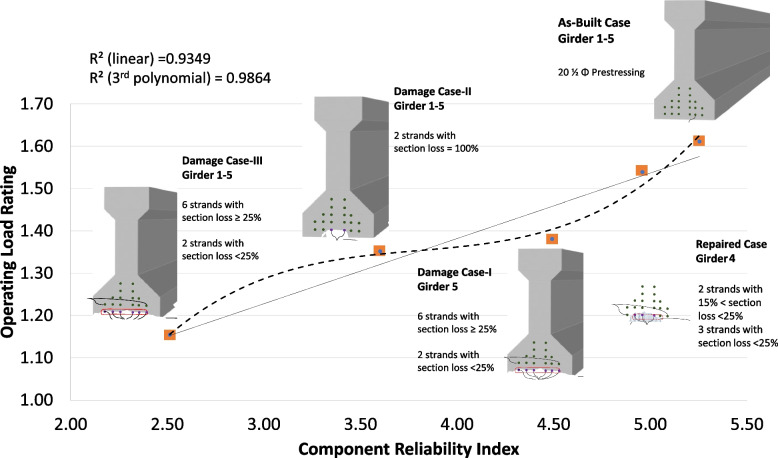
The relationship between operating load rating and component reliability is approximated using linear and 3.^rd^-degree polynomials for each case. A larger-scale representation of the controlling girders was previously presented in Figs. 5, 6, 7, 8 and 9

Figure 15 illustrates the controlling girder in each case presented on the operating load rating versus component reliability chart. A linear and a polynomial correlation can be observed between the operating load rating (OP LR) and component reliability index (βc) at different condition cases of the bridge span. Equation 15 and Eq. 16 are simple equations developed for this bridge, given the different conditions defined for this bridge. These equations can only provide a rough correlation estimate between load rating and reliability using the linear and 3^rd^-degree polynomial curves. The case study bridge has over 100 identical spans in real life. Equation 15 and Eq. 16 could be used to “estimate” load rating and component reliability at any of these spans based on conditions provided in these several cases.

15 $$OP LR=0.1548 \beta c+0.7635$$

16 $$OP LR = 0.0696 \beta c^3-0.7932 \beta c^2 +3.0522 \beta c-2.6104$$

It is also worth noting that the relationship between the component reliability and operating load rating is highly dependent on the uncertainty parameters utilized in the computation process of the reliability index. More information on the relationships between the reliability index and operating load ratings can be found in Gokce et al. (2011). While load rating is a good indication of the reliability of the bridge, it is not the only factor. For instance, the COV of the load rating distribution also has a significant correlation with the reliability index. This is understandable because higher COV for load rating distribution indicates higher uncertainty. Damage-I and Damage-II cases demonstrate this phenomenon. While the mean operating load rating of the Damage-II case is only 2.2% higher than the Damage-I case (1.38 vs 1.35), the increase in component reliability index is much higher at 20.3% (from 3.61 to 4.53). Damage-I investigates an actual damage case where a high uncertainty exists in estimating the cross-section loss in prestressing strands, while Damage-II investigates a hypothetical case of deterministic strands cross-section loss.

In addition, the results demonstrate that performing the field re-tensioning repairs not only increased the capacity of the girders (load rating) but also decreased the rate of uncertainty. Both factors contribute to increasing the component reliability and, consequently, the system reliability of the superstructure. This can be illustrated by examining the improvement from Damage-I (real damage case) to the Repaired cases. While the operating load rating increased by 11% (from 1.35 to 1.52), the COV decreased by 18% (from 14.2% to 11.6%). The two factors combined correlate with an increase in component reliability of 26%. It is worth noting that performing the repairs shifted the critical component from Girder 5 to Girder 4 (3.61 to 4.85), as shown in Table 5.

System reliabilities of Damage-I and Damage-III cases illustrate the significance of considering system reliability when assessing damaged/repaired bridge superstructures. When considering only load rating or component reliability calculations, the Damage-III case would be much worse than Damage-I. The mean operating load rating and the component reliability values for the Damage-III case are 1.15 and 2.52, while for Damage-I, they are 1.35 and 3.61, respectively. The system reliability for the Damage-III case is 3.23, and for the Damage-I case is 3.61. As a result, the component reliability of the Damaged-III case is 31% lower than the Damaged-I case. On the other hand, the system reliability of the Damaged-III case is only 11% less than the Damaged-I case. This is because the critical component in the Damage-III case is Girder 3; in the Damage-I case, it is Girder 5. This indicates that Girder 3 has more redundancy in the system reliability model than Girder 5, which improves the system reliability. This improvement is not shown in component reliability calculations but can easily be quantified by considering the system's reliability.

The main advantage of using the AASHTO load rating is that it is a standardized process. Since load and resistance factors are well-established in the code, analysis conducted by multiple engineers will produce close results for the same load, condition, and section properties. One drawback of using the AASHTO load rating as the sole evaluator for repaired bridges, as highlighted by the results of this paper, is its inability to capture all the uncertainties within the input parameters. For example, uncertainty in the remaining cross-section for prestressing strands is a big factor. It was shown here that the reliability of the bridge is not only related to the load rating but also to the load rating distribution (both mean and COV).

Component reliability, however, accounts for the added uncertainties in repaired bridges. System reliability accounts for the added uncertainties and redundancy, or lack thereof, offered to the member controlling the limit state. The disadvantages of reliability analysis are the increased complexity of calculations, the large amount of input data that may or may not be available, and the ability to influence the results by manipulating the input data (Estes and Frangopol 2005). For instance, while the bias factor of measured truck load responses to convert to 75-year maximum moments is reported by Nowak and Rakoczy (2013) to range from 1.3 to 1.5, significantly lower reliability will result from changing the bias factor from 1.3 to 1.5. Another disadvantage of reliability calculations is that real-life data may be scarce, limited, or not exists (Akiyama et al. 2020). In this paper, a literature review was utilized to address the lack of some data, such as material properties and load demand uncertainties, while field observation was utilized to make assumptions for other data, such as the correlation between resistance components and the extent of deterioration uncertainties.

## Summary and conclusions

The external impacts, such as environmental or man-made stressors, deteriorate prestressed concrete bridge girders over time, lowering their safety and serviceability. Although various structural repairs can be implemented to improve the carrying capacity of the structure, the introduction of unknowns during the repair process leads to high uncertainty in predicting the sufficiency of the repairs. Thus, this study investigates the effect of re-tensioning repairs of the prestressed concrete bridge girders (AASHTO II) on the component, system reliability, as well as load-rating values for the as-built, damaged, and repair scenarios while accounting for the uncertainties involved, such as structural repair (strand splicing), material properties and external loads. On this subject, the distribution for structural demand accounting for uncertainty in loads and the distribution for structural capacity accounting for uncertainties in material properties and repair process is defined for each girder in a span of the prestressed concrete bridge. Some case-specific and general conclusions are made of this study regarding the uncertainties in structural repair, material properties and external loads, and their effect on load rating and component and system reliability of the prestressed concrete bridge structure girders.

The case-specific conclusions of the study are presented below.

- Performing the structural repairs significantly improves both reliability factors and the load rating but does not wholly restore as-built conditions. Previous literature (Harries et al. 2012; Zobel and Jirsa 1998; Gangi et al. 2017) recommended using a deterministic value of the effective number of strands restored by splicing to be 85% of their original number. This study investigated the additional uncertainty in section loss in the spliced and exposed strands due to previous corrosion. New equations are introduced to account for these uncertainties.
- A damaged girder has a higher probability of failure (lower reliability index) due to the reduced capacity mean values and the higher uncertainty. Equations 11 and 12 offer a new approach to quantifying the mean reduction and higher uncertainty.
- Load rating distribution better indicates the component reliability than the load rating alone. For instance, Damage-II's mean operating load rating is only 2.2% higher than Damage-I's. However, the component reliability index increase is much higher at 20.3%. The higher rate of increase in component reliability could be understood from the lower COV of load rating distribution in the Damage-II case. It could not, however, be understood from the mean load rating alone.
- Increased uncertainty ranges increase the coefficient of variation in a load rating distribution and reduces the component reliability. Equations 9, 10, 11 and 12 provide a novel approach to quantify this uncertainty range in damaged and repaired prestressed girders by utilizing field-observed conditions to calculate the mean and standard deviations of the remaining strand cross-section.
- Performing repairs on a prestressed concrete bridge increases the reliability of the bridge components at a higher rate than the mean load rating, as the repairs reduce the uncertainty of the remaining capacity. The variation in the rate of increase between load rating and component reliability is case-by-case as it depends on the amount of uncertainty reduced by performing the repairs. By calculating the COV, Eq. 12 quantifies the uncertainty range in the remaining strands cross sections before the repairs, while Eq. 10 quantifies their uncertainty range after the repairs. For the repaired case studied in this paper, the mean operating load rating increased by 12% while the component reliability increased by 27%.
- System reliability directly accounts for the redundancy of the controlling girder in the system reliability model. In an S-P system, the location of the girder controlling the load rating and/or component reliability affects the system's reliability. This was illustrated in this paper. The component reliability of the Damaged-III case is 31% lower than the Damaged-I case, while the system reliability of the Damaged-III case is only 11% less than the Damaged-I case. Based on the system reliability model, the controlling girder in the Damage-III case, Girder 3, has more redundancy than in the Damage-I case, Girder 5.

The general conclusions of the study are presented below.

- Corrosion of strand damage introduces a new uncertainty in estimating the remaining cross-section of strands. While the AASHTO load rating process does consider the condition through factors, it is not case specific. In this paper, Equations were developed to quantify the uncertainties due to damage and repairs of the strands. Quantifying these uncertainties aid in the process of calculating load rating distribution and component and system reliabilities.
- Investigating the component reliability enhances the damage/repair assessment by considering the additional uncertainties introduced by the damage and during repairs.
- Generating a system model and calculating the system reliability gives the evaluating engineer a new tool to optimize the evaluation/rating process of damaged/repaired bridge superstructures by considering the amount of redundancy available to the controlling component.

## Data Availability

Some or all used models, codes, and detailed results are available from the authors of this paper upon request.
